# Several Distinct Polycomb Complexes Regulate and Co-Localize on the *INK4a* Tumor Suppressor Locus

**DOI:** 10.1371/journal.pone.0006380

**Published:** 2009-07-28

**Authors:** Goedele N. Maertens, Selma El Messaoudi-Aubert, Tomas Racek, Julie K. Stock, James Nicholls, Marc Rodriguez-Niedenführ, Jesus Gil, Gordon Peters

**Affiliations:** 1 Cancer Research UK, London Research Institute, London, United Kingdom; 2 Cell Proliferation Group, MRC Clinical Sciences Centre, Faculty of Medicine, Imperial College, Hammersmith Campus, London, United Kingdom; Roswell Park Cancer Institute, United States of America

## Abstract

Misexpression of Polycomb repressive complex 1 (PRC1) components in human cells profoundly influences the onset of cellular senescence by modulating transcription of the *INK4a* tumor suppressor gene. Using tandem affinity purification, we find that CBX7 and CBX8, two Polycomb (Pc) homologs that repress *INK4a*, both participate in PRC1-like complexes with at least two Posterior sex combs (Psc) proteins, MEL18 and BMI1. Each complex contains a single representative of the Pc and Psc families. In primary human fibroblasts, CBX7, CBX8, MEL18 and BMI1 are present at the *INK4a* locus and shRNA-mediated knockdown of any one of these components results in de-repression of *INK4a* and proliferative arrest. Sequential chromatin immunoprecipitation (ChIP) reveals that CBX7 and CBX8 bind simultaneously to the same region of chromatin and knockdown of one of the Pc or Psc proteins results in release of the other, suggesting that the binding of PRC1 complexes is interdependent. Our findings provide the first evidence that a single gene can be regulated by several distinct PRC1 complexes and raise important questions about their configuration and relative functions.

## Introduction

Polycomb Group (PcG) proteins, so named because of mutations that affect the patterning of the male sex combs in *Drosophila*, are transcriptional repressors that participate in distinct multiprotein complexes, the best characterized being Polycomb repressive complexes 1 and 2 (PRC1 and PRC2) [1], [2]. The PRC2 complex has three core components and catalyzes the trimethylation of histone H3 at lysine 27 (H3K27me3) [3] whereas the prototypic PRC1 complex comprises stoichiometric amounts of Polycomb (Pc), Posterior sex comb (Psc), Polyhomeotic (Ph) and Sex combs extra (Sce) [4], [5]. PRC1 binds to the H3K27me3 mark via the chromodomain of the Pc protein [6], [7] and catalyzes the mono-ubiquitylation of histone H2A on lysine 119, thereby shutting down transcription [8], [9] reviewed in ref. [2].

In mammalian cells, the situation is complicated by the presence of multiple orthologs of the archetypal PRC1 proteins. With five Pc proteins (CBX2, CBX4, CBX6, CBX7 and CBX8), six Psc proteins (BMI1, MEL18, MBLR, NSPC1, RNF159 and RNF3), three Ph proteins (HPH1, HPH2 and HPH3) and two Sce proteins (RING1 and RING2) there is enormous scope for combinatorial diversity [10], [11]. The reasons for such diversification and the interplay between the different family members remain unclear. Genetic ablation of specific PcG genes in mice has confirmed their role in embryonic patterning and *Hox* gene regulation but also pointed to more general effects on stem cell function. For example, *Bmi1* null mice have hematological and neurological defects that are traceable to a failure in the self-renewal of the relevant stem cells [12]–[15]. More recently, genome-wide ChIP analyses have identified over 1000 genes that are potential targets of PcG-mediated repression, many of which are implicated in the maintenance of pluripotency [16]–[18].

The hematological and neurological defects observed in *Bmi1* null mice can be largely rescued by concomitant ablation of the *Ink4a/Arf* tumor suppressor locus [12]–[15]. The locus encodes two unrelated proteins, p16^INK4a^ and p14^ARF^ (p19^Arf^ in mice), that activate the retinoblastoma and p53 tumor suppressor pathways, respectively (reviewed in [10], [19]. Both are implicated in cellular senescence, the state of permanent growth arrest that occurs when primary cells are exposed to various forms of stress, such as oncogenic signaling, telomere erosion or oxidative damage. Curiously, their impact appears to be context dependent with *INK4a* playing the predominant role in human cells and *Arf* assuming greater significance in mouse cells [10], [19].

Two strands of evidence point to an important role for Polycomb complexes in regulating the locus. Several mouse PcG gene knockouts have been shown to cause premature senescence in the derived embryo fibroblasts, due to derepression of *Ink4a/Arf* [20]–[25]. Conversly, ectopic expression of Bmi1, Cbx7, and Cbx8 has been shown to delay senescence in both mouse and human fibroblasts by downregulating *INK4a* [26]–[29]. As part of a broader effort to characterize the entire range of PRC1 complexes that regulate *INK4a* in human cells, we have identified MEL18 as a direct repressor of the locus in partnership with either CBX7 or CBX8. ChIP analyses revealed that CBX7, CBX8, MEL18 and BMI1 are all present at the promoter and first exon of *INK4a*, and shRNA-mediated knockdown of each protein causes upregulation of p16^INK4a^. Importantly, we present evidence that these proteins participate in multiple, distinct PRC1 complexes, that several PRC1 complexes bind simultaneously to the *INK4a* locus, and that their binding appears to be interdependent. To our knowledge, this is the first indication that PcG-mediated repression requires cooperation between several distinct types of PRC1 complex.

## Materials and Methods

### Cell culture, viral transduction and transient transfection

Primary human fibroblasts (FDF and Hs68) and HEK293T cells were cultured in Dulbecco-modified Eagles medium (DMEM) supplemented with 10% fetal calf serum (FCS), 100 IU/ml penicillin, and 100 µg/ml streptomycin. Recombinant lentivirus and retrovirus vectors (10 µg) were packaged in 293T cells (3.3×10^6^ cells per 10 cm dish) by co-transfection with either 2 µg of pCG-VsVG and 8 µg pCMVΔ8.2 [30], or with 2 µg pCG-VsVG and 8 µg pCG-GagPol [31], using a standard calcium phosphate based protocol. The following day (17 h post-transfection) the cells were washed and the medium was reduced to 5 ml. Virus was harvested 12 h later, filtered through a 45 µm filter and either used directly or stored at −80°C. Fibroblasts (8×10^5^ cells per 10 cm dish) were infected with 1 ml of virus diluted 5-fold in complete medium. Retroviruses were used undiluted. Additional medium was added 8 h later and infected cells were selected in either puromycin (3 µg/ml), hygromycin B (25 µg/ml) or blasticidin (2.5 µg/ml) as appropriate. Cells were generally harvested 14 days post-selection. For co-precipitation experiments, 293T cells were transfected with a total of 20 µg of plasmid DNA (10 µg of each construct or empty vector) and cells were harvested 48 h later.

Cell proliferation assays were performed by staining viable cells with crystal violet, as previously described [32]. Five days post-selection, 5×10^3^ cells were plated out into each well of a 24-well plate and 6 wells were used per time point.

### Plasmids and shRNAs

pBabePuro-mCbx7-FLAG and pBabeBlast-mCbx8-HA were generated by cloning the full-length cDNAs of mouse Cbx7 and Cbx8 in frame with C-terminal FLAG and HA tags, respectively. The mouse Cbx7 cDNA was also used to construct the mCbx7-TAP fusion in pcDNA6. Human CBX7-FLAG, CBX7-HA, CBX8-FLAG and CBX8-HA were expressed from a pcDNA6 based vector (Invitrogen), by sub-cloning the full-length cDNAs of CBX7 and CBX8 in frame with the C-terminal FLAG or HA epitope. A similar strategy was used to construct pGM-MEL18-FLAG. BMI1-FLAG was expressed from a pQCXIP based vector (Clontech), by sub-cloning the BMI1 coding domain in frame with the C-terminal FLAG epitope. All plasmids were sequence verified.

The following Mission shRNA constructs were obtained from Sigma (BMI1 shRNA1, NM_005180.5-1061s1c1; BMI1 shRNA2, NM_005180.5-693s1c1; BMI1sh3, NM_0051805-922s51c1; MEL18 shRNA1, NM_007144.2-840s1c1; MEL18 shRNA2, NM_007144.2-867s1c1; CBX7 shRNA1, NM_175709.1-736s1c1; CBX7 shRNA2, NM_175709.1-153s1c1; CBX8 shRNA1, NM_020649.1-1183s1c1; CBX8 shRNA2, NM_020649.1-166s1c1).

### Quantitative reverse transcription and PCR

Total RNA was extracted using the Ultra Pure RNA extraction Kit (Roche). cDNA was generated from 0.5–1 µg of RNA using MultiScribe reverse transcriptase and random hexamer primers (Applied Biosystems). One fiftieth of the cDNA was used as a template for quantitative real-time PCR (q-RTPCR). PCR products were detected with POWER SybrGreen (Applied Biosystems). GAPDH was used as a loading control. For detection of the *INK4a* transcript, annealing and extension was done for 1 min at 66°C, while for other transcripts this step was performed at 60°C. Sequences of the primers can be found in Supplementary Table S1.

### Immunoprecipitation and immunoblotting

Cells were washed extensively in phosphate buffered saline (PBS) and lysed in five cell volumes of IP lysis buffer (1% NP40, 10 mM Tris.HCl pH 7.5, 150 mM NaCl, 5 mM EDTA, 30 mM sodium pyrophosphate, 50 mM sodium fluoride, 10% glycerol, Complete EDTA free protease inhibitor (Roche) and 1 mM PMSF) at 4°C. Extracts were centrifuged for 30 min at 4°C and pre-cleared with Protein G Sepharose. For FLAG-tagged proteins, extracts were mixed with anti-FLAG M2 coupled agarose (Sigma) for 3 h, washed extensively in IP lysis buffer and eluted in the same buffer supplemented with FLAG peptide. Endogenous MEL18 and CBX8 were precipitated with anti-MEL18 (Abcam, ab5267) bound to Protein G Sepharose (GE Healthcare), or rabbit anti-CBX8 (GALD, kindly provided by Kristian Helin) [27] bound to Protein A Sepharose (GE Healthcare).

For total protein analysis, cells were washed in PBS and lysed in RIPA buffer (20 mM Hepes pH 7.6, 300 mM NaCl, 0.01% NP40, 1% sodium deoxycholate. 0.1% sodium dodecyl sulphate (SDS), 2 mM EDTA, Complete EDTA free protease inhibitor (Roche), and 1 mM PMSF). Samples (25 µg) of total protein were separated by SDS-polyacrylamide gel electrophoresis gel (PAGE) in a 12% gel and transferred onto a nitrocellulose membrane. The following primary antibodies were used: mouse anti-BMI1 (ab14389, Abcam), goat anti-MEL18 (ab5267, Abcam), rabbit anti-CBX8 (Bethyl Laboratories), rabbit anti-CBX7 (ab21873, Abcam), mouse anti-p16^INK4a^ (JC8), mouse anti-RING2 and mouse anti-HPH2 (both kindly provided by Haruhiko Koseki), rabbit anti-β-tubulin (H-235, Santa Cruz) and horse radish peroxidase (HRP)-conjugated anti-FLAG M2 (Sigma). Donkey anti-rabbit HRP (GE Healthcare), sheep anti-mouse HRP (GE Healthcare) and rabbit anti-goat HRP (Dakocytomation) conjugated antibodies were diluted 1∶2000 and signals were detected by ECL (GE Healthcare). HRP conjugated anti-GAPDH antibody (ab9482, Abcam) was used as a loading control.

### Chromatin immunoprecipitation

ChIP assays were performed as described previously [33]. For sequential ChIP experiments, the chromatin was eluted in 1% SDS, 10 mM EDTA, 50 mM Tris.HCl pH 7.5 for 10 min at 68°C [34]. The eluted chromatin was divided into equal fractions, diluted 10-fold in dilution buffer (0.01% SDS, 1.1% Triton X-100, 1.2 mM EDTA, 16.7 mM Tris.HCl pH 8.0, 167 mM NaCl) and the second ChIP was performed as the first step. The CBX8 antibody used for ChIP was raised in rabbits against the synthetic “LAST” peptide [16]. The IgG fraction of the serum was isolated and further purified against the peptide. Other antibodies were as follows: rabbit anti-CBX7 (ab21873, Abcam), rabbit anti-MEL18 (Santa Cruz Biotechnology), mouse anti-BMI1 (AF27, kindly provided by Kristian Helin), mouse anti-GFP (clone 3E1), and rabbit anti-histone H3K27me3 (Upstate). For sequential ChIP using FLAG- and HA-tagged mCbx7 and mCbx8, the chromatin was first enriched by binding of the sonicated chromatin to anti-FLAG (Sigma) or anti-HA Sepharose beads (Covance). After extensive washes, the chromatin was eluted with the cognate peptide. The eluate was divided in equal fractions, diluted 10-fold in dilution buffer and the second ChIP was performed as in the first step. An irrelevant IgG was used as negative control. The immunoprecipitated DNA was quantified using real-time qPCR with the primer sets described in reference [35].

### Tandem affinity purification of polycomb complexes

A 293T cell line that stably expresses mCbx7-TAP was produced by transfection of *AhdI* linearized pcDNA6-mCbx7-cTAP using Lipofectamine 2000 (Invitrogen). After 48 h, the cells were trypsinized and plated at several dilutions in medium containing blasticidin (10 µg/ml). Single colonies were picked 20 d later. A single clone was selected in which the expression level was moderate and uniform within the clone, as determined by immunofluorescence. 293T derived cell lines that stably express MEL18-TAP or BMI1-TAP were described previously [36]. Purification of mCbx7-TAP followed the same protocol except that ZnCl_2_ was omitted from the buffers, NaCl was at a concentration of 150 mM and TEV cleavage was conducted at 4°C overnight. Following separation of the proteins on 4–12% Bis-Tris Novex gel (Invitrogen) in MOPS buffer, the gel was stained with Colloidal Blue (Sigma). Visible bands were excised and subjected to trypsin digestion in gel and liquid chromatography-tandem mass spectrometry (LC-MS/MS). Only those protein identified with Mascot scores above a cut-off value of 50 were considered potential partners of mCbx7.

## Results

### Human PRC1 complexes contain single representatives of the Pc and Psc families

A PRC1 type complex containing CBX8 has been shown to act as a repressor of *INK4a* transcription in human fibroblasts [27]. Purification and characterization of this complex revealed a number of other PRC1 components, notably the Psc homolog BMI1 and both Sce proteins (RING1 and RING2), but no other member of the Pc family was reported to be present. However, we and others have shown that another Pc homolog, CBX7, also associates with and represses *INK4a* [10], [37], [38]. With the aim of identifying the PcG proteins that functionally associate with CBX7, we performed tandem affinity purification of complexes based on mouse Cbx7 (mCbx7), because of its relative stability, as well as human BMI1 and the closely related Psc protein MEL18. In each case, the bait protein was tagged at its carboxy terminus with the classical TAP motif, comprising elements of calmodulin binding protein and protein A separated by a TEV cleavage site [39]. Purification was performed using HEK293T cells that were stably transfected with the relevant vectors. Purified complexes were fractionated by SDS-PAGE and following Coomassie staining of the gel (Supplementary Fig. S1), the bands were excised and subjected to trypsin digestion and mass spectrometric analyses. In each case, a parallel analysis was performed on equivalent bands from a TAP-only control. Functional appraisal of the BMI1 and MEL18 complexes has already been reported [36]. As summarized in Supplementary Figure S1, we did not detect other Pc proteins in the mCbx7 complex and found no evidence that other Psc proteins co-purified with either BMI1 or MEL18, in agreement with recently published findings from other groups [27], [40].

**Figure 1:**
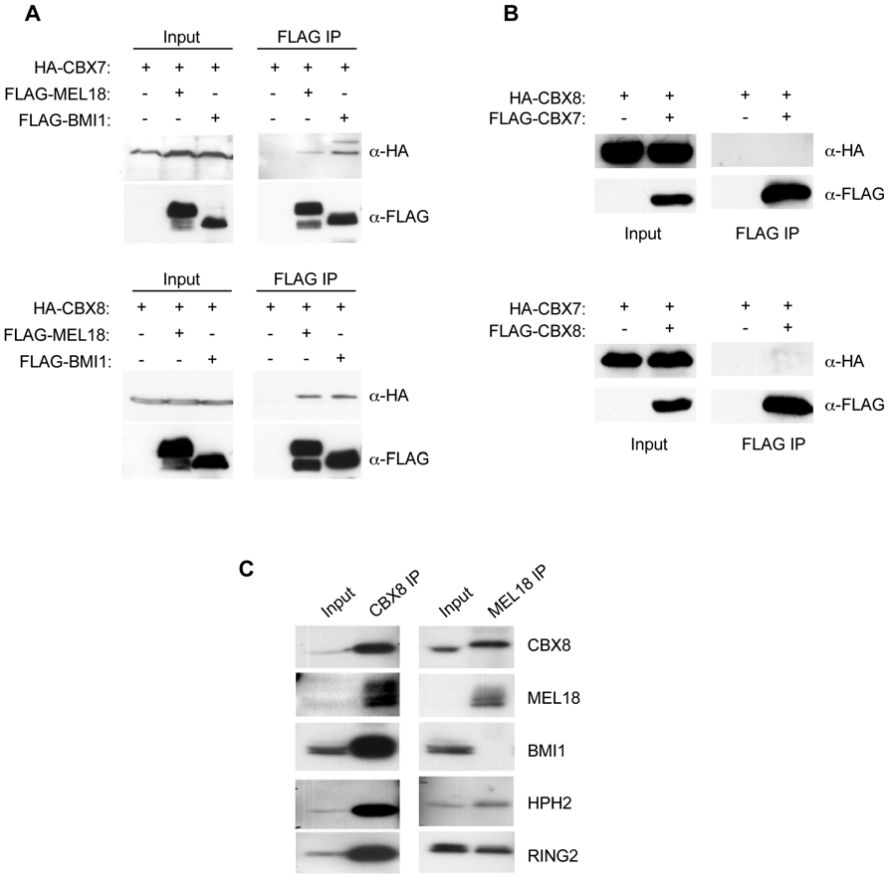
Interaction of CBX7 and CBX8 with BMI1 and MEL18. A. Co- immunoprecipitation of HA-CBX7 and HA-CBX8 with FLAG-BMI1 and FLAG-MEL18 from transiently transfected 293T cells. Left panels show direct immunoblotting of cell lysates. Right panels show the anti-FLAG precipitates immunoblotted for the respective FLAG- and HA-tagged proteins. B. Lack of interaction between CBX7 and CBX8 in 293T cells transfected with the respective HA- and FLAG-tagged proteins. C. Co-immunoprecipitation of endogenous PRC1 components, as indicated, using polyclonal antibodies against MEL18 and CBX8. Input shows direct immunoblotting of 5% of the sample used for immunoprecipitation.

As these conclusions relied on the absence of identifiable peptides in mass spectrometry analyses, we substantiated them in a number of ways. For example, when HA-tagged versions of human CBX7 and CBX8 were transiently expressed in 293T cells along with FLAG-tagged Psc proteins, it was possible to co-precipitate HA-CBX7 and HA-CBX8 with either FLAG-BMI1 or FLAG-MEL18 (Fig. 1A). However, when FLAG-tagged CBX7 was co-expressed with HA-tagged CBX8, there was no evidence for an interaction between these proteins and a similar result was obtained when the epitope tags were exchanged (Fig. 1B). Importantly, immunoprecipitation of endogenous MEL18 from 293T cells confirmed its interaction with CBX8 and lack of association with BMI1 (Fig. 1C). In a parallel experiment, we showed that BMI1 and MEL18 can both be co-precipitated with CBX8 antibodies. Thus far, we have been unable to extend these analyses to CBX7 because of interference from immunoglobulin bands on the western blots. Note that RING2 was also co-precipitated with MEL18 and CBX8 and the mass spectrometry data suggested that both RING1 and RING2 were present in all of the affinity purified complexes that we have characterized (Supplementary Fig. S1). In additional experiments, in which we conducted affinity purification of MEL18 in cells expressing FLAG-tagged RING1 and HA-tagged RING2, or vice versa, we demonstrated unequivocally that RING1 and RING2 are present in distinct complexes (Supplementary Fig. S2). Taken together, our data imply that human PcG proteins can participate in multiple PRC1-type complexes and that each complex contains single representatives of the Pc, Psc and Sce families.

### *INK4a* is regulated by multiple PRC1 components in primary HDFs

**Figure 2:**
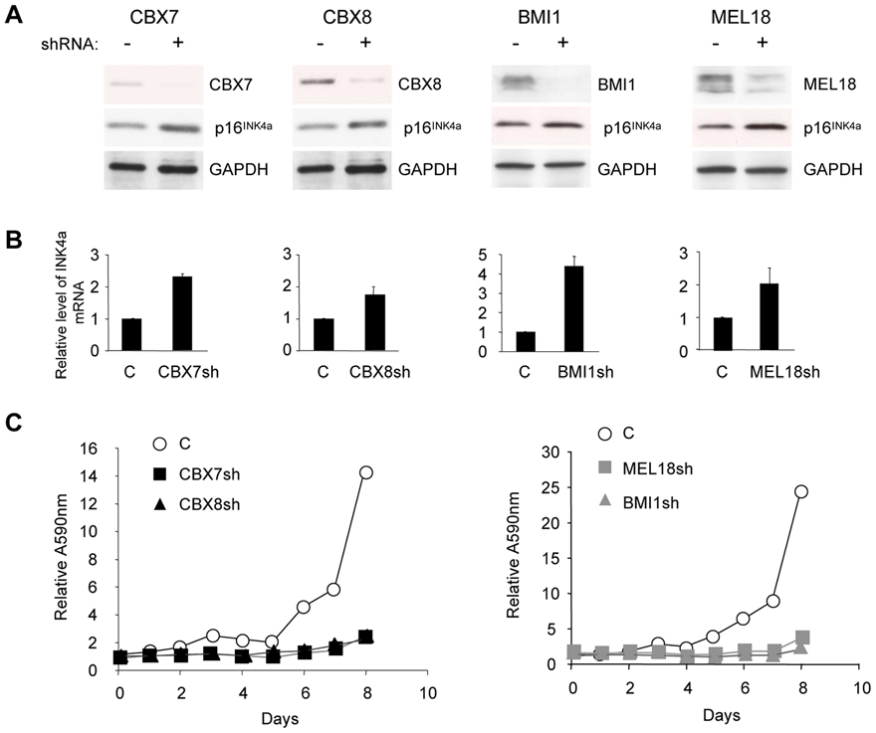
*INK4a* is regulated by multiple PRC1 proteins in human fibroblasts. A. FDF cells were infected with lentiviruses encoding shRNAs against CBX7, CBX8, BMI1, or MEL18, or an irrelevant control shRNA (C). Effects on the respective target proteins and on p16^INK4a^ were assessed by immunoblotting with the indicated antibodies. B. Corresponding changes *INK4a* mRNA were quantified by qRT-PCR. C. Effects of the indicated shRNAs on cell proliferation was assessed by crystal violet staining. Each time point represents an average of six replicates and the A_595nm_ values were normalized to the absorbance at day 0 (first day after plating).

Minimally, our data suggested that CBX7 and CBX8 form at least four distinct PRC1-type complexes with MEL18 or BMI1. It was therefore important to establish which of these combinations might be implicated in the repression of *INK4a* in human fibroblasts. To this end, we first asked whether shRNA-mediated knockdown of individual PRC1 components affected *INK4a* expression. After empirically testing multiple shRNAs against each target gene, we used lentiviral vectors to express the most effective hairpins in primary fetal dermal fibroblasts (FDF). The extent of knockdown was evaluated by qRT-PCR and immunoblotting. As exemplified in Figure 2, shRNAs against CBX7, CBX8, MEL18 or BMI1 each resulted in a significant increase in p16^INK4a^ expression at both the protein (Fig. 2A) and RNA levels (Fig. 2B). These effects were consistently observed with independent shRNAs but their magnitude varied depending on the strain of fibroblasts in which the experiments were conducted (Supplementary Fig. S3). As expected from the de-repression of p16^INK4a^, knockdown of each of the PRC1 components caused a significant impairment of cell proliferation culminating in a senescence-like growth arrest (Fig. 2C). The low levels of p14^ARF^ in primary HDFs precluded detection by immunoblotting but we did not observe a consistent change in ARF transcript levels following knockdown of PRC1 components (see below). At face value, therefore, our data support the idea that multiple PRC1 components participate in the repression of *INK4a* in human fibroblasts.

### MEL18 binds directly to the *INK4a* locus

**Figure 3:**
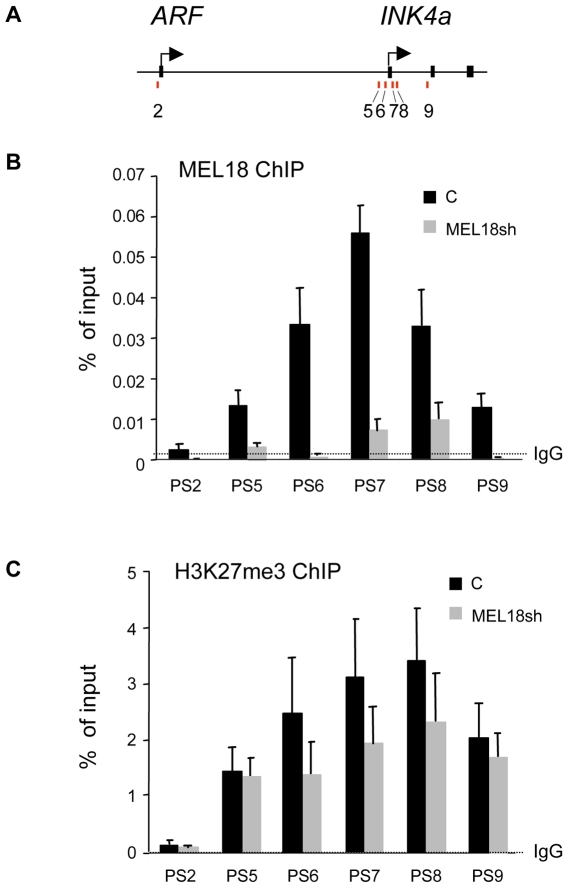
MEL18 binds directly to the promoter and first exon of INK4a. A. Schematic of the human *INK4a-ARF* locus showing the location of coding exons (black boxes) and transcription start sites, not drawn to scale. Numbered bars refer to the approximate locations of primer pairs used for PCR interrogation of precipitated chromatin (as described in ref. [35]. B. ChIP analyses of MEL18 binding across the locus in normal fibroblasts (black bars) or in cells that had been transduced with MEL18 shRNA (grey bars). An irrelevant IgG was used as a negative control. Results are presented as % of input DNA. C. Corresponding ChIP data for H3K27me3.

Although genetic ablation of the mouse *Mel18* gene has been shown to cause premature senescence and de-repression of *Ink4a/Arf* [23], there are conflicting views about its role in regulating the locus [41]. To try to clarify the situation, we performed ChIP analyses with previously validated primer sets (Fig. 3A and ref. [35] to establish whether MEL18 interacts directly with the endogenous human *INK4a* locus in normal fibroblasts. The pattern of MEL18 binding was very similar to that described for other PRC1 components and the H3K27me3 mark, with a peak centered on the first exon of *INK4a* and very little binding at the *ARF* promoter region (Fig. 3B and C). Specificity was confirmed by the fact that shRNA-mediated knockdown of MEL18 caused a marked reduction in the ChIP signal (Fig. 3B). Interestingly, the knockdown of MEL18 also resulted in a loss of H3K27me3 across the first exon of *INK4a* (Fig. 3C), suggesting that PRC1-type complexes contribute to the maintenance of the H3K27me3 modification. Alternatively, as components of the PRC2 complex are regulated by E2F transcription factors [42], loss of H3K27me3 might simply reflect impaired cell proliferation caused by derepression of *INK4a*.

### Role of BMI1 and MEL18 in mCbx7-mediated repression of *INK4a*

**Figure 4:**
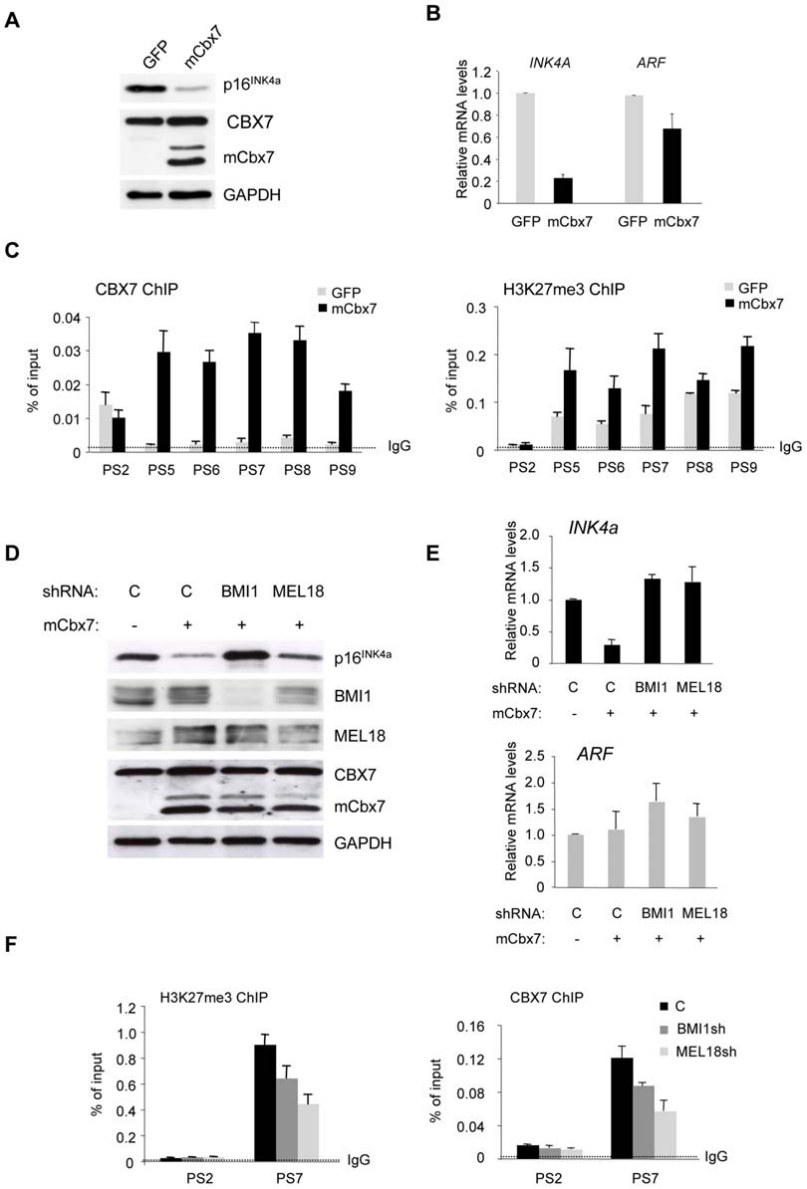
Involvement of BMI1 and MEL18 in mCbx7-mediated repression of *INK4a*. A. Immunoblot analyses of FDF cells infected with retroviruses encoding either mCbx7 or GFP (negative control) showing down-regulation of p16^INK4a^. B. qRT-PCR analyses of *INK4a* and *ARF* RNAs in cells described in panel A. C. ChIP analyses of CBX7 (mouse+human) and H3K27me3 levels across the *INK4a-ARF* locus in cells expressing GFP or mCbx7, using the indicated primer sets (as in Fig. 3). D. The mCbx7 expressing cells were infected with lentivirus-based shRNA vectors targeting BMI1, MEL18, or an irrelevant control (C). Effects on the respective target proteins and on p16^INK4a^ were assessed by immunoblotting with the indicated antibodies. GAPDH was used as a loading control. E. qRT-PCR analyses of *INK4a* and *ARF* RNAs in cells described in panel D. F. ChIP analyses of CBX7 (mouse+human) and H3K27me3 in cells expressing shRNAs against BMI1 or MEL18. Anti-rabbit IgG was used as a negative control. The analyses were confined to primers corresponding to the proximal part of INK4a exon 1α (PS7) and ARF exon 1β (PS2).

As a more cogent test of the functional relevance of each component, we next asked whether the ability of CBX7 to downregulate p16^INK4a^ was dependent on either BMI1 or MEL18. HDFs that had been stably transduced with mCbx7 were superinfected with lentiviral vectors expressing shRNAs against BMI1 and MEL18. Relative to the GFP control, mCbx7 caused a substantial reduction in p16^INK4a^ RNA and protein levels, as expected, whereas p14^ARF^ RNA levels were essentially unchanged (Fig. 4A and 4B). ChIP analyses confirmed that ectopic mCbx7 caused a substantial increase in CBX7/mCbx7 binding around the first exon of *INK4a*, detected using an antibody that recognizes both the mouse and human versions of the protein, with little if any change at the *ARF* promoter (Fig. 4C). This was accompanied by an increase in the H3K27me3 modification, particularly at positions coincident with CBX7 (Fig. 4C). Importantly, shRNA-mediated knockdown of either BMI1 or MEL18 restored p16^INK4a^ levels in the mCbx7-expressing cells, again without affecting ARF (Fig. 4D and 4E). Moreover, ChIP analyses revealed that the depletion of BMI1 and MEL18 led to a decrease in CBX7/mCbx7 binding and H3K27me3 at the *INK4a* locus (Fig. 4F). These findings suggest that BMI1 and MEL18 are rate limiting for repression of *INK4a* by mCbx7.

### CBX7 and CBX8 bind simultaneously to the same segment of the *INK4a* locus

**Figure 5:**
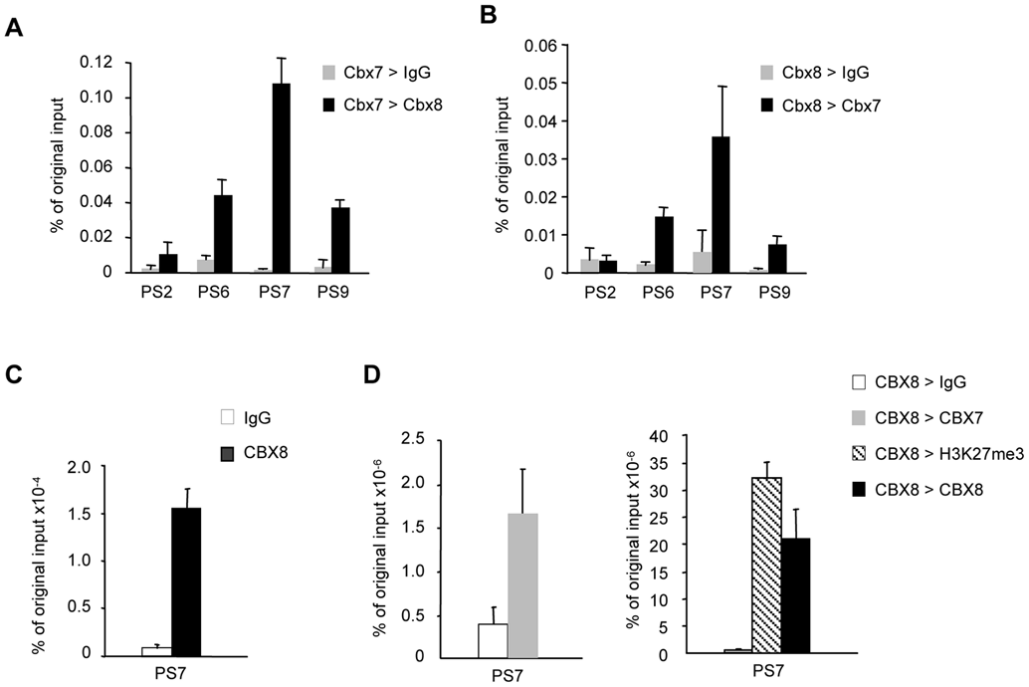
CBX7 and CBX8 bind simultaneously to the same *INK4a* allele. A and B. Sequential ChIP using HDFs that co-express FLAG-tagged mCbx7 and HA-tagged mCbx8. In A, the first ChIP was performed with the FLAG antibody, followed by either anti-HA or irrelevant IgG. In B, the first ChIP was performed with the HA antibody, followed by either anti-FLAG or IgG. The precipitated DNA was analyzed by qPCR with the indicated primer sets. The histograms represent the average and SD values of biological replicates. C and D, Sequential ChIP of endogenous CBX8 and CBX7 in FDF cells. Chromatin was first precipitated with affinity purified anti-CBX8 antibody and enrichment for *INK4a* primer set 7 (PS7) was confirmed by q-PCR. The chromatin was divided into four equal fractions and re-precipitated with either rabbit IgG as a negative control, anti-H3K27me3 or anti-CBX8 as positive controls, or anti-CBX7. The enrichment is calculated relative to the original input chromatin. The figure shows the results of a single representative experiment.

As we work with pools of cells, the ability of several PRC1 components to modulate *INK4a* expression is open to different interpretations. For example, it is conceivable that different PRC1 complexes regulate the locus in different cells or that several PRC1 complexes congregate at the locus in every cell. To try to distinguish between these possibilities, we set out to determine whether CBX7 and CBX8 can be detected on the same piece of DNA, using a ChIP-reChIP strategy. HDFs were co-infected with retroviruses encoding FLAG-tagged mCbx7 and HA-tagged mCbx8 to generate pools of drug-resistant cells expressing both proteins. An initial ChIP was performed using the FLAG antibody and the chromatin was eluted from the beads with the FLAG peptide. The recovered chromatin was then immunoprecipitated with the HA antibody or with an irrelevant IgG and subjected to qPCR analysis with a subset of the primer sets described in Figure 3A. The signal was quantified relative to the input material used in the first ChIP. As illustrated in Figure 5A, mCbx8 was substantially enriched at the *INK4a* locus following a mCbx7 ChIP. In the reciprocal experiment, the chromatin was first precipitated with the HA antibody and eluted with HA peptide. Again, it was clear that mCbx7 was enriched in the chromatin precipitated through mCbx8 (Fig. 5B), implying that mCbx7 and mCbx8 bind simultaneously to the same fragment of chromatin.

We were conscious that over expression of the mouse Pc proteins in HDFs could have created an artificial situation. In order to establish that human CBX7 and CBX8 can co-localize on *INK4a*, we performed sequential ChIP targeting the endogenous proteins. Affinity purified CBX8 antibody was used in the first step with a non-specific rabbit IgG as a negative control. After elution, a fraction of the precipitated chromatin was analyzed by quantitative PCR in order to determine the amount of CBX8 bound to *INK4a* in the first step (Fig. 5C). The recovered CBX8-bound chromatin was divided into four equal portions and immunoprecipitated with antibodies against CBX7, CBX8 and H3K27me3 or with a non-specific anti-rabbit IgG as a negative control. Due to the relatively small amounts of chromatin recovered after the sequential ChIP, the PCR analyses were restricted to primer pair PS7 which targets the proximal part of the *INK4a* exon 1α. Remarkably, the data clearly showed that endogenous CBX7 can co-precipitate with CBX8 on the same chromatin fragment (Fig. 5D). As expected, H3K27me3 also co-precipitated with the Pc proteins (Fig. 5D). Taken together, our data strongly suggest that CBX7 and CBX8, and by implication distinct PRC1 complexes, can bind simultaneously to the *INK4a* locus.

### Interdependence between PRC1 complexes for binding to the *INK4a* locus

**Figure 6:**
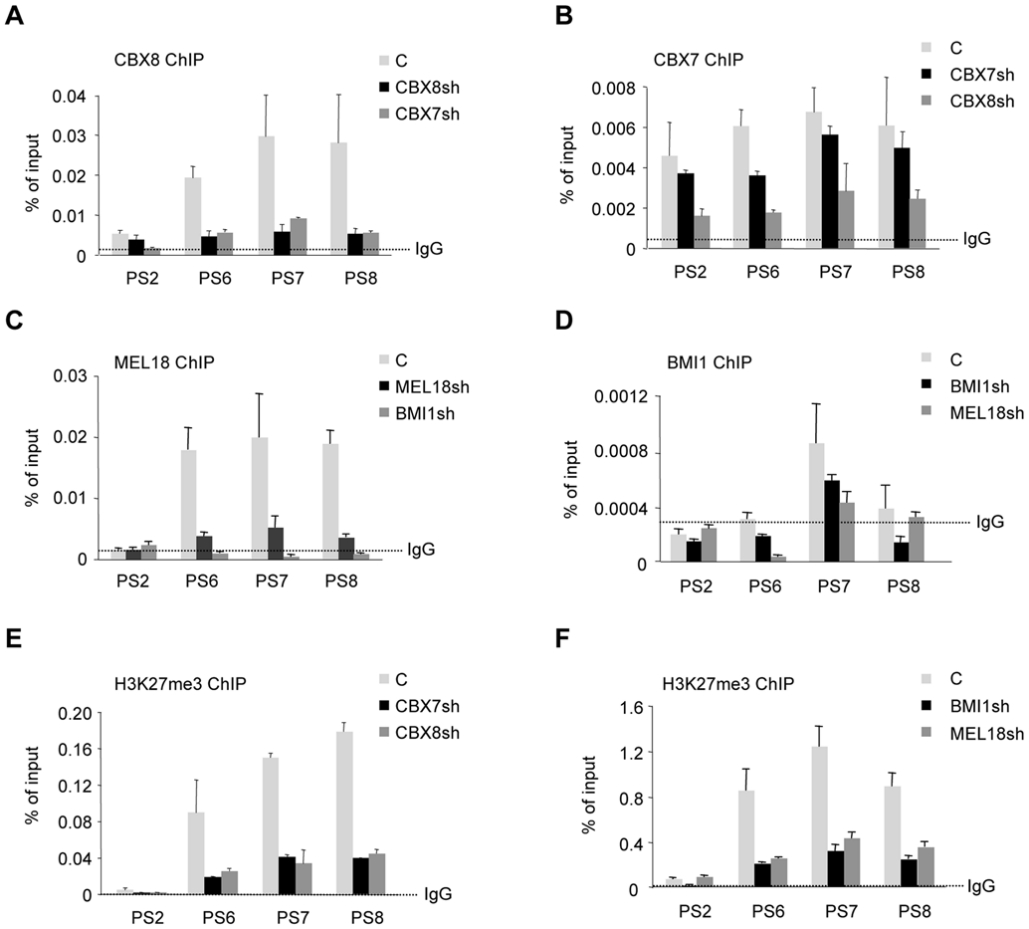
Interdependent binding of PRC1 components at the *INK4a* locus. ChIP assays were performed in FDF cells infected with lentivirus-based shRNA vectors targeting CBX8, CBX7, BMI1, MEL18, or an irrelevant control (C) as indicated. The chromatin was precipitated with rabbit antibodies against CBX8, CBX7, MEL18 and H3K27me3 or with a monoclonal antibody against BMI1. An anti-rabbit IgG was used as a negative control, except for BMI1 where the control was mouse anti-HA. The precipitated DNA was subjected to qPCR with the indicated primer sets (as described in Fig. 3) and the data are presented as percentage of input DNA.

At face value, the sequential ChIP data implied that distinct PRC1 complexes containing either CBX7 or CBX8 co-localize on a region of *INK4a* represented by primer sets 5–9. At the current level of resolution, it is not possible to judge whether specific complexes have preferred locations but this seems unlikely given the potential numbers of PRC1 complexes that might be involved. However, if all of these complexes are able to regulate the expression of *INK4a*, it is unclear why shRNA-mediated ablation of single components should result in de-repression. A pertinent observation was that when we knocked down the levels of endogenous CBX7 with shRNA, this led to a substantial decrease in the binding of CBX8 at the *INK4a* locus (Fig. 6A). Conversely, knockdown of CBX8 resulted in reduced binding of CBX7 (Fig. 6B). Along similar lines, depletion of BMI1 caused a concomitant loss of MEL18 at the locus and vice versa (Fig. 6C and D). These effects were not a consequence of cross-talk between the respective genes. For example, shRNAs against MEL18 had little if any effects on the expression of BMI1 and likewise knockdown of BMI1 did not cause significant changes in MEL18 RNA levels (Supplementary Fig. S4). Similar conclusions apply to CBX7 and CBX8 transcript levels. Interestingly, depletion of any one of these components resulted in a decline in H3K27me3 at the *INK4a* locus (Fig. 6D and E). Taken together, our findings imply interdependence between the multiple PRC1 complexes that bind to and regulate the *INK4a* locus and that the PRC1 complexes might contribute to the maintenance of the H3K27me3.

## Discussion

The data we present provide important and novel insights into the regulation of *INK4a* by PcG proteins and suggest a previously unrecognized level of complexity in PRC1 function. Although it is widely appreciated that mammalian cells express multiple orthologs of the prototypic Pc, Psc, Ph and Sce proteins, the potential for combinatorial specificity has not been properly addressed [10], [43]. There have been several previous descriptions of mammalian PRC1 complexes as well as of complexes reconstituted from recombinant proteins [8], [9], [27], [36], [40], [44]–[46]. Collectively, these leave open the possibility that, for example, both RING1 and RING2 may be present in the same complex and it is clear that some components can oligomerize [47], [48]. In an experiment specifically designed to ask whether RING1 and RING2 co-purify, we found no evidence that they were present in the same soluble complex (Supplementary Fig. S2) and our analyses of several affinity purified complexes and co-immunoprecipitation data (Fig. 1 and Supplementary Fig. S1) are consistent with the idea that each PRC1 complex contains a single representative from the Pc, Psc, Ph and Sce families, in line with the *Drosophila* paradigm.

It would be interesting to extend these analyses to obtain a more comprehensive picture of the possible combinations that exist in human cells but as proof of principle we focused on four components for which ChIP grade antibodies are available. Specifically, we have shown that the Pc homologs CBX7 and CBX8 can participate in PRC1-type complexes with at least two representatives of the Psc family, MEL18 and BMI1. All four of these proteins contribute to the regulation of *INK4a* as demonstrated by shRNA-mediated knockdown experiments (Fig. 2 and Supplementary Fig. S3). For CBX7, CBX8 and BMI1, this is consistent with published findings [23], [27], [28] but our study is the first to compare the different proteins within the same cell system. In the case of MEL18, there have been conflicting views about its effects on *INK4a* and cell proliferation. While many regard MEL18 as a functional analog of BMI1, with overlapping but distinct activities [23], [36], [40], [49]–[51], others consider MEL18 as a sequence-specific DNA binding protein that negatively regulates the expression of *BMI1* through an ability to repress *MYC* [41], [52]. In this scenario, MEL18 and BMI1 are inversely correlated and have opposing effects on *INK4a*. While there are precedents for cross-regulation of PcG genes [16], [53] and for a role for MYC in the regulation of *BMI1* [54], these ideas are difficult to reconcile with the fact that MYC can also activate *INK4a* in human cells [55] and that *BMI1* and *MYC* collaborate in tumorigenesis [56], [57]. It is possible that different PcG proteins might be rate limiting in different cell types, or that MEL18 could influence cell proliferation or differentiation independently of its effects on *INK4a* [41], [58], [59]. However, we find that in primary human fibroblasts, knockdown of MEL18 with several independent shRNAs results in de-repression of *INK4a* and premature senescence without appreciable changes in BMI1 expression. We also present compelling evidence that MEL18 binds directly at the *INK4a* locus with a similar distribution to that of BMI1 and other PRC1 proteins.

Genetic ablation of several PcG genes, including *Mel18*, *Bmi1*, *Ring1b* and *Mph2*, results in a skeletal transformation and stem cell defects in mice, and curtailed lifespan in the corresponding MEFs [20]–[25]. Although *Ink4a* expression is clearly deregulated in these MEFs, *Arf* plays a more prominent role in senescence in mouse cells and in some cases the effects of the PcG knockout can be alleviated by concomitant inactivation of *Arf* [24], [60], [61]. In human cells, however, it is not clear that p14^ARF^ levels are affected by or contribute significantly to senescence [62], [63] and it has proved difficult to demonstrate that ARF is subject to regulation by PcG complexes [27], [35], [64], [65]. Consistent with this, we found that the levels of H3K27me3 at the *ARF* locus were very low in normal human fibroblasts and that *ARF* expression was generally unaffected by experimental modulation of PRC1 components.

As well as highlighting the role of MEL18, our data imply that several distinct PRC1 complexes are capable of regulating *INK4a*. Moreover, sequential ChIP experiments based on CBX7 and CBX8 suggest that these complexes are present on the same region of chromatin. Perhaps the most remarkable aspect of our findings is that knockdown of any one component caused de-repression of *INK4a* and loss of H3K27me3, suggesting some degree of cooperation or interdependence. While it is feasible that loss of one component or PRC1 complex could expose the locus to other influences, such as H3K27 histone demethylase activity, it is not clear why other complexes are unable to substitute. One possibility would be that each complex binds at a specific location but this seems unlikely considering the number of potential permutations associated with the locus. Within the limited resolution of standard ChIP protocols, the PRC1 components showed the same relatively narrow peak of binding around the promoter and first exon of *INK4a*, as described in previous studies, with H3K27me3 showing a broader distribution [27], [35], [64], [65]. A more plausible explanation would be that the complexes form a higher order structure. This would explain the pleiotropic effects of individual shRNAs and why it is difficult to quantify the relative importance of individual PRC1 proteins.

Although expression profiling and genome wide ChIP on chip studies have implied that PcG genes may be subject to transcriptional regulation by PRC1 complexes [16], we did not find consistent evidence for such effects between the components studied here. Moreover, the prediction would be that loss of one PcG protein might enhance the expression of another, the converse of the interdependence we observe. However, it is clear that their participation in a higher order structure could present a complicated picture in terms of the influence of one protein on the stability or post-translational modification of another. Indeed, we noted that although ectopic expression of mCbx7 had no effect on BMI1 and MEL18 RNA, there was a significant increase in the respective protein levels (Fig. 4D).

At this juncture, we have no information on how many complexes bind to the locus and what sort of higher order complexes might be involved but there are obvious precedents for long-range effects and chromosome looping in PcG-mediated repression [66], [67]. An aggregate of PRC1 complexes might be required to form or stabilize the loop and in principle our data do not exclude association between different alleles. The ability of some components to oligomerize, notably the Ph family members [47], [48], could facilitate interactions between neighboring complexes. Alternatively, complexes might be brought together by an RNA component, and again there are several precedents and possibilities, including short interfering RNA or antisense non-coding transcripts [68]–[72]. Based on current understanding, PRC1 complexes have the potential to bind to H3K27me3 and RNA, and to ubiquitylate H2A [6], [8], [9], [36]. However, a requirement for several distinct PRC1 complexes in the regulation of *INK4a* implies that the complexes might have distinct properties or functions. In this context, it will be important to establish whether *INK4a* represents a special case or whether other PcG target genes are similarly regulated by multiple types of PRC1 complex.

## Supporting Information

Table S1 Table of primers used
(0.06 MB DOC)

Figure S1 Analyses of affinity purified PRC1 complexes. A. SDS-PAGE analyses of proteins that co-purify with TAP tagged mCbx7, MEL18 or BMI1 from 293T cells. The gels were stained with Colloidal Coomassie Blue and photographed. Visible bands were subjected to trypsin digestion and tandem MS/MS analyses. In each case, control bands were excised from a parallel purification using the TAP vector alone. The named proteins on the right of each gel identify known PcG proteins for which signature peptides were identified by mass spectrometry. B. A diagrammatic summary of known PcG proteins recovered in each purification. Note that the circled bait proteins are the only representatives of their family detected in the respective complex.
(0.11 MB TIF)

Figure S2 RING1 and RING2 are present in distinct complexes. HEK293T cells that stably transduced with TAP-tagged MEL18 were co-transfected with plasmids encoding FLAG-tagged RING1 and HA-tagged RING2 (middle panel) or HA-tagged RING1 and FLAG-tagged RING2 (right panel). The MEL18 complexes were affinity purified according to the schedule on the left. Following TEV cleavage, to release the complexes from the IgG beads, the complexes were subjected to a second round of affinity purification on anti-FLAG beads. This effectively recovers a complex of MEL18 and one of the FLAG tagged RING proteins. The recovered material was then eluted with FLAG peptide, fractionated by SDS-PAGE, and immunoblotted for the FLAG, HA and CBP epitopes, as indicated. The asterisk in the right panel indicates a non-specific band which co-purified in this schedule. Importantly, HA-tagged RING2 did not co-purify with MEL18 and FLAG-tagged RING2 and likewise, HA-tagged RING1 did not co-purify with MEL18 and FLAG-tagged RING2.
(0.08 MB TIF)

Figure S3 Derepression of INK4a with different PcG shRNAs in different fibroblast strains. The figure shows several experiments that re-capitulate the effects documented in Figure 2, namely that shRNA-mediated knockdown of CBX7, CBX8, BMI1 and MEL18 results in up-regulation of p16INK4a at the protein (left panels) and RNA levels (right panels). For each PRC1 protein, the effects could be observed with at least two independent shRNAs and in several strains of human fibroblast. The data for CBX8, BMI1 and MEL18 refer to Hs68 cells.
(0.06 MB TIF)

Figure S4 Lack of cross talk in the regulation of PRC1 gene expression. A. Knockdown of CBX7 with independent shRNAs had little if any effect on the expression of CBX8 and vice versa as assayed by qRT-PCR. B. Similarly, shRNAs against BMI1 had negligible effects on MEL18 and vice versa. C. In cells over-expressing mCbx7 (as described in Figure 3), knockdown of BMI1 had no effect on MEL18 and vice versa.
(0.05 MB TIF)
