# Supplementary material for: Several Distinct Polycomb Complexes Regulate and Co-Localize on the INK4a Tumor Suppressor Locus
Source: PLoS One. 2009 Jul 28;4(7):e6380. doi: 10.1371/journal.pone.0006380 (PMC2713427; doi:10.1371/journal.pone.0006380)
Supplement: Table S1 — Table of primers used (0.06 MB DOC) [file pone.0006380.s001.doc]

**Table S1**. **Sequences of the oligonucleotide primers used for qRT-****PCR.**
