# Supplementary figures and images for: Several Distinct Polycomb Complexes Regulate and Co-Localize on the INK4a Tumor Suppressor Locus
Source: PLoS One. 2009 Jul 28;4(7):e6380. doi: 10.1371/journal.pone.0006380 (PMC2713427; doi:10.1371/journal.pone.0006380)

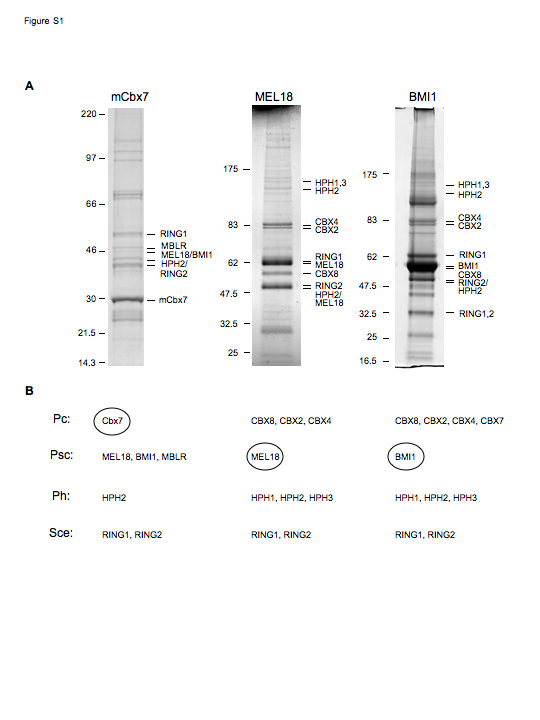

Supplement: Figure S1 — Analyses of affinity purified PRC1 complexes. A. SDS-PAGE analyses of proteins that co-purify with TAP tagged mCbx7, MEL18 or BMI1 from 293T cells. The gels were stained with Colloidal Coomassie Blue and photographed. Visible bands were subjected to trypsin digestion and tandem MS/MS analyses. In each case, control bands were excised from a parallel purification using the TAP vector alone. The named proteins on the right of each gel identify known PcG proteins for which signature peptides were identified by mass spectrometry. B. A diagrammatic summary of known PcG proteins recovered in each purification. Note that the circled bait proteins are the only representatives of their family detected in the respective complex. (0.11 MB TIF) [file pone.0006380.s002.tif]

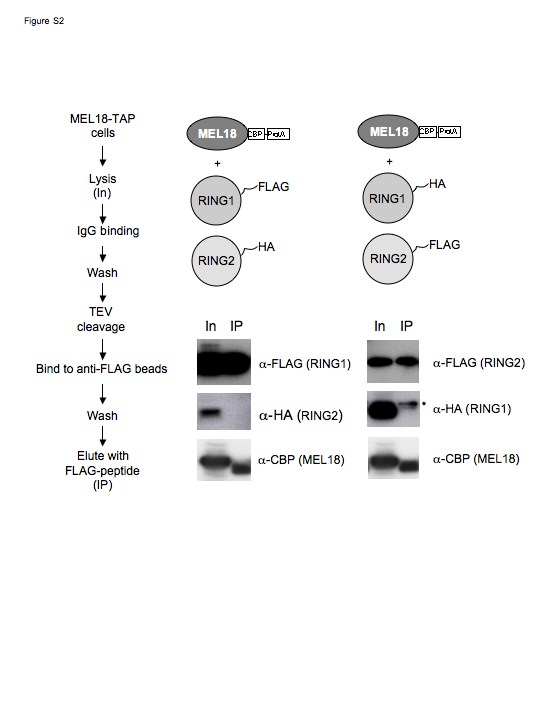

Supplement: Figure S2 — RING1 and RING2 are present in distinct complexes. HEK293T cells that stably transduced with TAP-tagged MEL18 were co-transfected with plasmids encoding FLAG-tagged RING1 and HA-tagged RING2 (middle panel) or HA-tagged RING1 and FLAG-tagged RING2 (right panel). The MEL18 complexes were affinity purified according to the schedule on the left. Following TEV cleavage, to release the complexes from the IgG beads, the complexes were subjected to a second round of affinity purification on anti-FLAG beads. This effectively recovers a complex of MEL18 and one of the FLAG tagged RING proteins. The recovered material was then eluted with FLAG peptide, fractionated by SDS-PAGE, and immunoblotted for the FLAG, HA and CBP epitopes, as indicated. The asterisk in the right panel indicates a non-specific band which co-purified in this schedule. Importantly, HA-tagged RING2 did not co-purify with MEL18 and FLAG-tagged RING2 and likewise, HA-tagged RING1 did not co-purify with MEL18 and FLAG-tagged RING2. (0.08 MB TIF) [file pone.0006380.s003.tif]

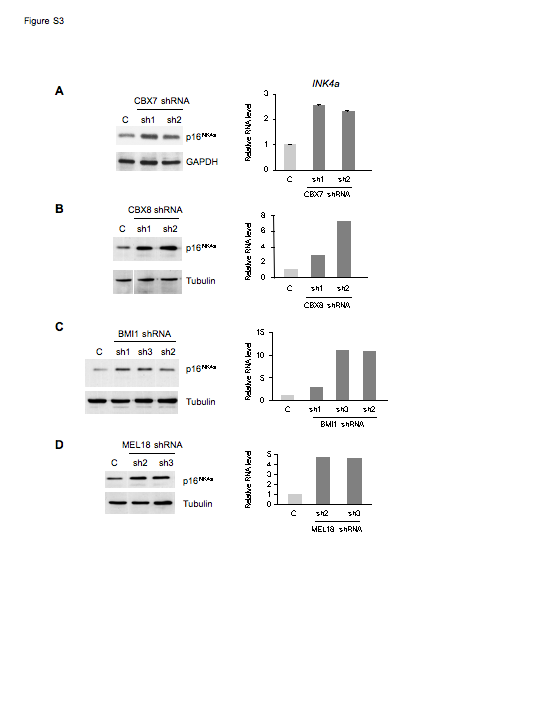

Supplement: Figure S3 — Derepression of INK4a with different PcG shRNAs in different fibroblast strains. The figure shows several experiments that re-capitulate the effects documented in Figure 2, namely that shRNA-mediated knockdown of CBX7, CBX8, BMI1 and MEL18 results in up-regulation of p16INK4a at the protein (left panels) and RNA levels (right panels). For each PRC1 protein, the effects could be observed with at least two independent shRNAs and in several strains of human fibroblast. The data for CBX8, BMI1 and MEL18 refer to Hs68 cells. (0.06 MB TIF) [file pone.0006380.s004.tif]

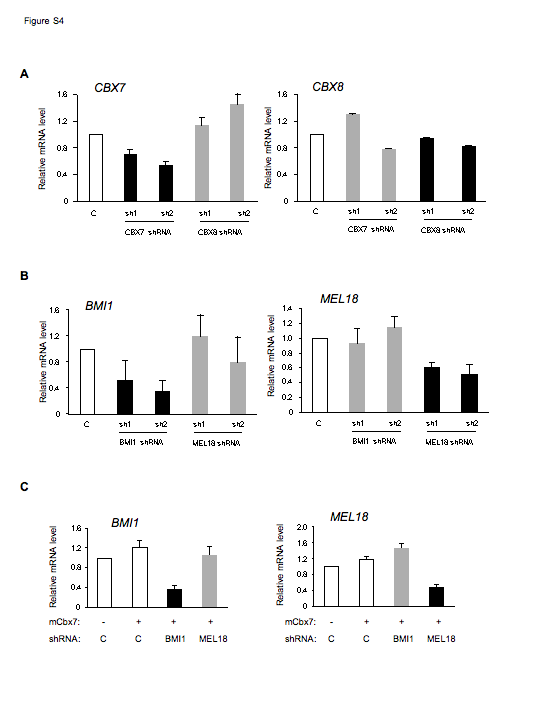

Supplement: Figure S4 — Lack of cross talk in the regulation of PRC1 gene expression. A. Knockdown of CBX7 with independent shRNAs had little if any effect on the expression of CBX8 and vice versa as assayed by qRT-PCR. B. Similarly, shRNAs against BMI1 had negligible effects on MEL18 and vice versa. C. In cells over-expressing mCbx7 (as described in Figure 3), knockdown of BMI1 had no effect on MEL18 and vice versa. (0.05 MB TIF) [file pone.0006380.s005.tif]
